# Case report: A dual case study of how clinical feedback can be a communication aide and influence therapeutic work

**DOI:** 10.3389/fpsyg.2023.1199431

**Published:** 2023-12-13

**Authors:** Marianne Magnesdotter Helleseth, Andrew Athan McAleavey, Christian Moltu

**Affiliations:** ^1^District General Hospital of Førde, Førde, Norway; ^2^Department of Health and Caring Sciences, Western Norway University of Applied Science, Bergen, Norway

**Keywords:** case study, clinical feedback, psychotherapy, psychotherapeutic alliance, routine outcome monitoring

## Abstract

**Background:**

While routine outcome monitoring and clinical feedback may improve outcomes after psychotherapy, results from efficiency studies have been mixed. Moreover, how clinical feedback is implemented influences how it works for patients and clinicians, and working mechanisms are hitherto not thoroughly explored. Researchers have argued that inviting and using feedback from patients is best conceived of as a clinical skill. In this paper, we use case study methodology to explore and describe feedback’s functions within three clinical skill themes: actualizing alliance work, concretizing change and stagnation and verbalizing the non-verbal.

**Case presentation:**

Sonja is a young adult patient with a trauma background. She has a history of serious suicide attempts and distrust in relationships. She attended psychotherapy for eight months. Harald is a middle-aged man with a stable family. Traumatic events in his past has made him conceal own needs and developing depression. He attended psychotherapy for 19 months. Case material include the patient’s clinical feedback over a range of life areas, medical health notes and the therapist’s process notes.

**Conclusion:**

Clinical feedback can be a positive supplement to the therapeutic work and process. The importance of making this as a joint tool between the client and the therapist is significant.

## Background

Routine outcome monitoring with immediate clinical feedback (ROM-CF) can improve the effectiveness of treatment for the therapist and patient (Lambert and Shimokawa, 2011). However, systematic reviews show that these effects are not uniform, and the current opinion is that they might help some patients in some contexts, rather than all patients in all contexts (McAleavey and Moltu, 2021).

In a scoping review, Krägeloh et al. (2015) reported that the integration of structured and formalized ROM-CF with clinical practice had a significant impact on their effectiveness. In short, when such systems are used as part of clinical communication, they seem to be beneficial; however, they have limited clinical benefits when they are implemented for other purposes in an organization. There is an emerging set of findings suggesting that this may be because the participants in treatment must actively use ROM-CF, often in multiple ways, to generate positive results, so that simply adding a measure and offering feedback is not sufficient to improve treatment outcomes. Several recent qualitative studies have found that the process of using ROM-CF in a helpful way is best understood as a clinical skill requiring active participant involvement, from the perspectives of both the therapist (Hovland et al., 2020) and client (Solstad et al., 2020a,b). These found that positive experiences with ROM-CF generally involve active use of the data in treatment, with collaborative meaning-making processes between therapists and patients a key part of the process. Consistent with this perspective, Brooks Holliday et al. (2021) have suggested best practices for the clinical use of measurement-based care, including the clinical process of repeating rationales, engaging clients in meaning-making and concretizing problems and changing processes via visual presentation.

In a recent commentary, McAleavey and Moltu (2021) cautioned against overly simplistic interpretations of ROM-CF. While it is important to know whether there is an average treatment effect on outcome from ROM-CF, the mechanisms of ROM-CF are likely complex and dyadic. They suggested that ROM-CF be studied as ‘an accelerator for treatment personalization: a tool to help patients and therapists adjust, modify, and improve their care’ (p 143). They described ROM-CF as a dyadic and contextual process. This position echoes earlier observations that for clinical feedback to be effective, it must be aligned with the client’s and the therapist’s needs and be relevant to the way they work together (Bickman et al., 2000; Bickman, 2008). This position has implications for what is important to research. Understanding ROM-CF calls for inquiry into what the dyadic processes are, and how they influence individual therapy. In addition to studying the outside effects of add-on interventions, we must acquire experiential knowledge about the underlying mechanisms of the intervention, to guide future hypotheses.

As a widely adopted strategy with limited evidence, ROM-CF in psychotherapy needs a practice-research investigation into the processes of its use. The systematic case study with multiple sources of information is one strategy to acquire first-hand knowledge of complex phenomena (McLeod and Elliott, 2011).

In the present study, we used a dual case study approach to explore the research question: Which clinical processes are influenced by the use of structured feedback from the client to enhance or support clinical conversations?

## Case presentation

### Methods

In this section, two clinical cases are presented along with information about the therapist. The identities of the clients who contributed to this article were anonymized using pseudonyms to protect their privacy. Both clients consented to participate in the experiences and processes that were part of this report, and both of them read and commented on the interpretation and presentation of the data. Their perspectives are presented in the Results section.

### Clients and the therapist

#### Client A

At intake, Sonja was formally assessed in line with national guidelines, including clinical interviews, structured diagnostic interviews and brief symptom measures. She was diagnosed with major depressive disorders, with a previously diagnosed borderline personality disorder assessed to have remitted into subclinical current presentation. Sonja is a young girl who was sexually molested as a child. Her experience resulted in a painful state of mind and several suicide attempts. At the start of the therapeutic relationship, she had multiple admissions to the in-patient clinic and one previous experience of long-term therapy. Due to her history of traumatic experiences, she had difficult relationships with significant others. She never felt understood and found it hard to achieve a good connection with her family. This left her with a sense of emotional distance from them, which resulted in lack of trust in their ability to reconnect as a family. Despite this difficulty, she met and developed a relationship with a man who became her boyfriend. At the time she starting therapy, they had settled and established a good home-life together, where honesty and open communication were important values of their relationship. Together with her boyfriend, Sonja enjoyed and cultivated healthy interests. At the same time, she struggled with a prolonged challenge of a poor level of care for her own basic needs. This made it difficult for her to be as active as she wanted to be involved in local community affairs or to assume the role she desired as a volunteer. When starting therapy, Sonja presented with great sorrow over her lost youth. She wanted help with painful experiences and difficult emotions. She struggled with shame, and due to her relational experiences, it was hard for her to open up for this work in therapy. Although she found it very difficult, she attended almost every session and made great efforts during the sessions. Throughout her time in therapy, Sonja made great strides. When the therapy ended, Sonja reported a reduction in her experiences of inner pain. She had worked on her basic needs, her emotional struggles, and importantly, she had managed to find a stable job, where she still works and enjoys it. Therapy with Sonja lasted eight months.

#### Client B

At intake, Harald was formally assessed in line with national guidelines, including clinical interviews, structured diagnostic interviews and brief symptom measures. He was diagnosed with post-traumatic stress disorder. Harald is a middle-aged man with a well-established life. He has a wife, children, close friends and good neighbors. He finds himself to be the person who always offers a helping hand, often sacrificing his own needs to fulfill the wishes and requests of others. Harald felt a pronounced need to do this, due to his personal history. Harald carried with him a traumatic experience that haunted him. Several decades ago, he was responsible for a serious accident, which resulted in a short prison term. He felt the need to pay back by helping others and thereby experience forgiveness. Harald did not speak aloud about the accident, or his ongoing struggle with it that still affected his everyday life. Harald had never asked for psychological help before, and therapy was a new experience for him. He was not accustomed to talking about his feelings and tended to avoid disclosing how he really felt. He struggled with anxiety, depression, low self-worth and shame, but most of all an inability to forgive himself. Due to this, he felt the need to protect his loved ones from his inner self, which resulted in an attempt to hide his pain, making him feel distant and alone. Throughout the therapy, Harald re-established a more open and connected relationship with his significant others and became more forgiving and accepting of himself. The therapy with Harald lasted 19 months.

#### The therapist

The therapist for these two clinical cases was a psychologist from Norway. She had worked in different areas of the mental health field, but during Sonja’s and Harald’s therapy sessions, she worked in an outpatient clinic on the west coast of Norway. Throughout her career, she applied training in emotion-focused therapy, which had a clear impact on how she met her clients and her approach to their challenges. She had a special interest in the relational aspects of therapy and the emotional components of the therapeutic process. At the same time, she was involved in the development of the clinical feedback system Norse Feedback while she maintained her clinical practice. She had played an active role in the training and supervision of other therapists using feedback systems in general and Norse Feedback in particular. This knowledge of clinical feedback systems, along with her theoretical point of view, in all probability, influenced her clinical assessments and choices during the course of therapy with the two clients presented in this study.

### Routine outcome monitoring and clinical feedback

The ROM and clinical feedback systems have been available for clinicians for a long time, and their use is increasing in mental health services (Solstad et al., 2020b). Norse Feedback, a digital, multidimensional system (Moltu et al., 2018; Hovland et al., 2020), is a second-generation feedback system, which was developed in Health Forde, Norway, and built to meet the needs and wishes of patients and clinicians. It includes a broad range of issues that could be relevant to the patient, and it allows the patient to give feedback on a wider range of their everyday lives, and therefore, includes not only questions about mental health symptoms and recovery, but also about resources and interpersonal and social role dimensions. Norse Feedback includes 90 items and more than 17 dimensions (readiness to recovery, social safety, recovery environment, sad affect, somatic anxiety, trauma reaction, eating problems, substance use, substance recovery, avoidance (situational, social and interpersonal), self-criticism, hopelessness, worry, irritability, control, general functioning and cognitive difficulties) and therapy preferences and alliance formation (McAleavey et al., 2021).

Before the first meeting, the patient receives a short text message with a link to the clinical feedback on their mobile phones. The link is personalized to provide data-secure personal access to their specific form. Based on data and automated analyses, Norse Feedback transfers their answers to a clinical feedback report, and the report is automatically available to the clinician. Based on the methodology, the feedback system learns which areas and dimensions are significant for individual patients, and irrelevant areas are eliminated. Due to a trigger item, the system is sensitive to reported changes, and the areas can re-open in reaction to a client’s responses. In this way, the clinical feedback system adapts to changes in the client, and thereby provides a feedback system that is more personalized (Solstad et al., 2020a; McAleavey et al., 2021; Moltu et al., 2021).

**FIGURE 1:**
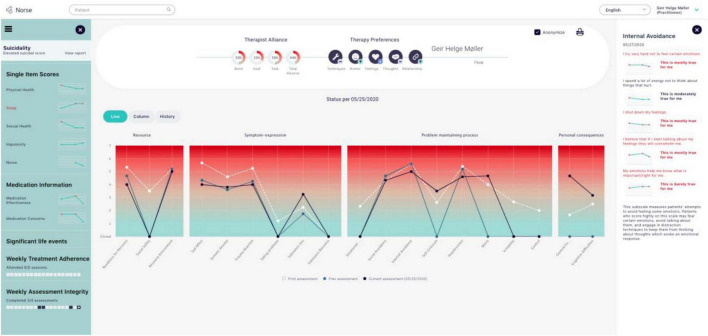
A clinician’s report, the main overview/dashboard screen monitored by therapists using the Norse Feedback. Specific aspects of this Figure are expanded for legibility in later figures.

In order for patients to experience ROM-CF as a useful tool, it is important that the therapist who receives the clinical feedback is sensitive to the patient’s individual needs and preferences, makes the experience meaningful to the patient and uses the feedback in a flexible way (Solstad et al., 2020b). Norse Feedback continuously develops to fit the needs of its users, through regular revisions (Moltu et al., 2021). Figure 1 shows an example of a clinician’s Norse Feedback report.

The Norwegian Mental Health Service a public entity that is decentralized and available through specialist mental health centers, i.e., distriktspsykiatriske senter (DPS). All residents of Norway have free health care, and mental health care is part of the specialist health service. The staff in the mental health service includes clinical specialists in psychology and psychiatry, psychologists and medical doctors and specialized clinical nurses and social workers. The DPSs include both bed-units and outpatients clinics. In the outpatient clinics, it is common for each therapist to have approximately 30–40 patients for whom they are responsible for providing treatment and follow-up. The bed-units provide specialized care from practitioners from different fields, as well as services that are more general, ranging from acute care to long-term treatment. Collaboration is common between units, thereby preventing gaps in patients’ treatment.

### Study design

There are various approaches to acquiring an in-depth understanding of different phenomena. The case study method provides opportunities to view the object of research from different perspectives. The purpose of the case study is to explore various aspects of the phenomenon studied and create meaning from them (McLeod and Elliott, 2011). What makes the case study an especially valuable approach is the possibility of taking a closer look at the complexity of the interactions within phenomena. This method may include the influence of contextual variables on the studied phenomenon and changes over time. In this study, we have purposefully selected two parallel cases for analysis, within a naturalistic/systematic case study approach (Iwakabe and Gazzola, 2009). Systematic case studies aim at thick descriptions and triangulation of different sources of data. The choice of a dual case analysis in our study is not primarily driven by comparison, but by variation, reflecting knowledge that the phenomenon in question is not working uniformly across people and contexts (McAleavey and Moltu, 2021). By thick descriptions of two processes in which different aspects of clinical feedback emerge as important we hypothesize that a more contextual and dyadic understanding of the phenomenon can result. Case studies can be helpful for revealing nuance in clinicians’ therapeutic understanding, and therefore, can be particularly relevant to clinical practice (McLeod and Elliott, 2011). The findings of case studies in psychotherapy can also have a sensitive dimension as they often include insights from several different perspectives on the same therapeutic processes (Bernhardt et al., 2021). We based this study on our clinical work with two clients at an outpatient clinic in Norway.

### Data material

The therapist wrote a clinical note in the health record after every session. These were primary data material, together the feedback reports generated by Norse Feedback (see Figure 1) for both patients. The therapist’s personal process notes were also available for analysis. On average, these notes were about 200 word each, comprising the parts 1. Status, 2. Progress, 3. Session focus. Additional information from patients’ prior medical records was included as contextual data. A member-checking procedure for the findings was implemented to ensure we stayed true to the lived experiences of the participants throughout the data analysis, and their feedback is reported in this study.

Sonja’s therapy involved 17 meetings and 13 clinical feedback reports. Harald’s therapy consisted of 44 meetings and 33 clinical feedback reports. We have numbered the medical health records (therapist session notes) and clinical feedback reports (Norse Feedback) throughout the study for easier referencing. The medical health record is numbered as HRA1-17 for Sonja and HRB1-44 for Harald. The clinical feedback report is numbered as FRA1-13 for Sonja and FRB1-33 for Harald. Table 1 provides an overview of the data material included in the study.

**TABLE 1:** Overview of the included data material.

Data material	Sonja	Harald	Total number
Medical health records	17	44	61
Clinical feedback reports	13	33	46
Therapy process notes	13	28	41

### Data analysis

The data material were analyzed within the framework of a team-based structured thematic analysis (Braun and Clarke, 2006). Because of the authors’ close proximity to the data (i.e., the first author being the therapist and the other authors being part of the team that researched and developed the Norse Feedback system), care was taken to ensure their transparency and reflexivity (Maso, 2003; Binder et al., 2012). First, the first author collected and prepared all of the data material for the analyses, which included an in-depth reading of the data in its entirety while taking preliminary notes of meaningful themes. Second, the first and last authors met for a first analytic seminar to go through the material and the first author’s preliminary notes. Based on this meeting, the data material was sorted into three preliminary themes: (a) relationship issues, (b) therapeutic processes and (c) difficulties. Third, the first author sorted the data material by preliminary themes, and discussed the data that did not fit with any of the themes with the last author. A wide range of different data fit with the theme of therapeutic processes, indicating that this thematic formulation was too broad to be useful. Fourth, the first author coded the data using a bottom-up approach by selecting chunks of data under each theme and assigning them a descriptive tag. At this stage, 27 descriptive tags resulted. One example of such a tag was a session note coded with the tag “Stagnation.” Fifth, the first and last authors reviewed all the codes and revised the thematic descriptions to encompass their abstract meanings across the descriptive tags. At this stage, the overarching themes were reformulated to include: (a) alliance formation, (b) change and stagnation and (c) verbalization processes. Twenty of the 27 descriptive tags from stage four fitted withing the stage five themes, and seven were discarded as idiosyncrasies that we did not make sense of across the two cases. Sixth, the first author wrote a coherent presentation of the themes’ content, using illustrations from both cases to describe meanings and variations in the themes. Seventh, the Results section was given to the two clients for member checking, and their feedback and perspectives were recorded and are presented in this study. Finally, all three authors shared their input and comments related to the overall presentation of the findings, with the second author assuming the role of commentator and questioning the process of analyses from the perspective of an outsider.

## Results

In light of our research question, “which clinical processes are influenced by the use of structured feedback from the client to enhance or support clinical conversations,” three themes emerged from the analysis of the data material across the two cases. Although manifestation of the themes differed slightly between the dyadic cases, we found that all three were importantly present in both cases. Importantly, the therapist for the two participants was the same; therefore, the results might be biased due to the therapist’s influence on the process, which is natural and would be expected. However, the themes found resonance in the individual stories of the two patients. The themes generated by the analyses were: (a) feedback actualized the work of alliance formation, (b) feedback concretized change and stagnation and (c) feedback helped to verbalize the non-verbal.

### Feedback actualized alliance work

This theme covered ways that feedback related to the therapeutic alliance. Working on the alliance was experienced as powerful and intimidating by both clients. The use of clinical feedback in the therapy sessions with Sonja and Harald was helpful for navigating the work on alliance formation directly. The process notes suggest that the therapist paid attention to the alliance scores on multiple occasions and aimed to direct attention to them during the clinical dialogue. The clinical health record shows that these dialogues, which occurred on multiple occasions, influenced the focus and intensity of the therapeutic work.

#### Sonja

Due to Sonja’s attitude that people could not be trusted, she struggled in the initial phases of therapy. Based on the knowledge acquired through conversations with Sonja and her clinical feedback, the therapist found it especially important to focus on the establishment of a therapeutic alliance. By being transparent and working to establish a safe therapeutic atmosphere, the process notes suggest that the therapist aimed for Sonja to experience the situation as sufficiently safe so that she could be open to difficult experiences. However, we can see in the feedback report that this focus did not have a particularly effective influence on the work of alliance formation (Supplementary Figure 1).

This feedback with the low scores on all the alliance concepts (task, goal, bond and total) suggests that Sonja did not feel safe and did not understand the purpose of the session. The medical record from this session states: ‘*we used this session for Norse Feedback. I showed the client the clinical report and we talked about the information. The client was positively involved in the interpretation and understanding of the report’* (HRA3). In this example, we can see that the information from the clinical feedback report resulted in concrete actions from the therapist in the form of explicit conversations about the alliance. Based on this intervention, the therapist and Sonja developed a joint understanding of how to comprehend the information in the feedback report and how it could influence the relationship between the client and the therapist in the future. At the same time, these initial explorations provided the client with a rationale for giving feedback continuously and with the knowledge that this feedback would be given weight in the process.

Sonja found the use of clinical feedback to be helpful for more easily sharing what was painful for her. She told the therapist that it made it possible for her to signal her difficulties through other channels of direct dialogue. At the same time, this information made it easier for the therapist to capture Sonja’s unspoken needs. The therapeutic contact between Sonja and the therapist gradually became stronger. The medical health record suggests that to a greater extent, Sonja dared to express what she thought and felt about the therapist and the clinical work: ‘*The client said at the end of the session that she does not find the sessions to be useful when she feels emotionally disconnected, as she did today. I tried to normalize this experience, and emphasize that “it is okay; this also happens in our sessions*”’ (HRA8). Having looked at continuous clinical feedback from Sonja, the therapist knew that Sonja had extensive struggles with worry and internal avoidance. Based on this information, the therapist was able to work on the best way to listen to this utterance in a sensitive and therapeutic manner. The therapist chose to reflect on how this experience could be viewed in the broader context of her struggles, and how the therapist could include this more explicitly in her therapy. Together, with information on symptom expression, it was more likely for the therapist to face this sensitive moment in a constructive way so that Sonja would hopefully feel taken care of and not ruminate because of the statement. The medical health record from the next session showed that Sonja dared to disclose what she felt to an even greater extent: ‘*The client said that she thought a lot about our last session. She did not think this session was useful, (.), but she did not dare explain the reason why in the last session. She said that she did not feel safe with me. She felt that I was too gentle and that she needed me to be less gentle in some situations’ (*HRA9). The therapist chose to let this be a focus during the therapy session, and together with Sonja, they explored how this need could be understood and met. The clinical feedback report that follows this session (Supplementary Figure 2) shows that the therapeutic work between the client and the therapist resulted in an immediate change in the feedback report from Sonja.

Information from the clinical feedback reports helped the therapist to balance the therapeutic interventions based on how Sonja felt. Having the latest information, the therapist had the opportunity to be more sensitive to unforeseen events and Sonja’s emotional state of the day. The process notes suggest that this focus had an impact on the overall therapeutic relationship between Sonja and her therapist. The medical health record from the latter session has the following statement: ‘*I informed her that I will be changing my job. She started to cry and said that this made her very sad’* (HRA11). The focus on in-depth conversations and work related to the therapeutic relationship and processes had a positive impact on the connection between Sonja and the therapist. The clinical feedback report conveys a visual assumption that the client also felt this connection (Supplementary Figure 3).

#### Harald

Most of the time during therapy with Harald, the therapist considered him to give good and friendly contact. The therapist stated the following in the health record: ‘…*he speaks easily and his contact with others is good’* (HRB2). At the same time, the therapist had information from the clinical feedback report that Harald perceived their alliance as weak, as evidenced in the ninth feedback report (Supplementary Figure 4).

This feedback remained unchanged for several months, and the process notes indicate that the therapist had numerous considerations about the need to achieve a joint understanding of the difficulties in the work of alliance formation and the stagnation in therapy between herself and Harald. According to the therapist’s note in the health record, ‘…*we spent most of the session on Norse Feedback, especially the alliance and needs in therapy’* (HRB12). The therapist tried to establish a common understanding of the emotional pain and the therapeutic work that was needed, but Harald did not experience the results of this effort as either clarifying or understandable. He reported having a weak alliance, which indicated that he did not experience what the therapist tried to convey as right or useful to him. The clinical feedback during the remainder of this conversation showed that the client never reported a strong alliance, despite the recent attempts to connect (Supplementary Figure 5).

This feedback suggested to the therapist that it would not be helpful to continue the therapy without elaborating on the rationale for the interventions and establishing a collaborative relationship with Harald. The report in the medical health record from this period of therapy states, ‘…*we talk about how this could provide a better understanding of the treatment plan, cf Norse’s goals and tasks’* (HRB16). This conveys the impression that the therapist and Harald had seemingly found a joint focus for the following sessions. Even though they seemed to agree on this, Harald continued to report struggles with the alliance in the clinical feedback report (Supplementary Figure 6).

The unchanged feedback on the alliance was again explored with Harald. The health record states, “*the client started the session with reflections on his feedback. I decided to print the feedback to make this a visual tool for us. The client told me that it is difficult for him to express his needs in therapy due to a fear of disappointing me. We spent time on the psychoeducation, based on Norse and how we could understand this fear, based on the client’s story’* (HRB27). The clinical feedback from the following session indicates that this conversation was of therapeutic importance, and it had a direct impact on the feedback (Supplementary Figure 7).

Based on the clinical feedback from Harald, we can see that he now starts to express needs and preferences in therapy through the feedback report, which signaled a wish for a higher level of a certain intervention. This was the first time Harald had expressed this kind of need during the feedback. Due to this experience, the therapist suggested increasing the frequency of therapy to weekly sessions. We can see in the feedback report that after the increase, Harald almost immediately resumed his reporting difficulties with the alliance (Supplementary Figure 8).

This change in the reported alliance was important, and the therapist used the session to work on this issue: “*he said that he was overwhelmed after the last session because of the focus we had (*…*). He told me that he would like to continue this process, but he feels anxious and tries to avoid the interventions. He said that he was afraid that I did not think he was doing a good enough job in the sessions and that he was afraid that the treatment would be terminated’* (HRB31). From the therapist’s perspective, the clinical feedback allowed the therapist to capture and verbalize Harald’s unspoken withdrawal from her. This information guided the therapist toward the important process of meaning making with Harald. According to the therapist’s report in the health record, ‘…*Harald was surprised over how effective the intervention had been and how difficult it had been to avoid the feelings. He felt that I (the therapist) came into ‘the innermost room’ – the room nobody should enter. He became restless and ashamed because of this. We spend a lot of time on this (*…*)’* (HRB31). The clinical health records indicate that Harald verbalized his difficulties directly with the therapist to a greater extent. Harald contributed retrospective reflections on the situation, making it easier to work on this issue.

### Feedback concretized change and stagnation

This theme related to the impact that feedback had on showing the participants change and the lack thereof. It can be difficult for patients to notice small signs of improvement, especially when change is gradual. Receiving continuous feedback from a client gives the therapist an opportunity to use the clinical feedback report with the client, and, in doing so, have the advantage of using the visual changes recorded in the report as evidence of the client’s motivation to persevere with the therapeutic process. Feedback systems are thus one way to identify gains that might otherwise be hard to observe. Visualizing changes may also be relevant when there is stagnation or in cases of worsening.

#### Sonja

**FIGURE 2:**
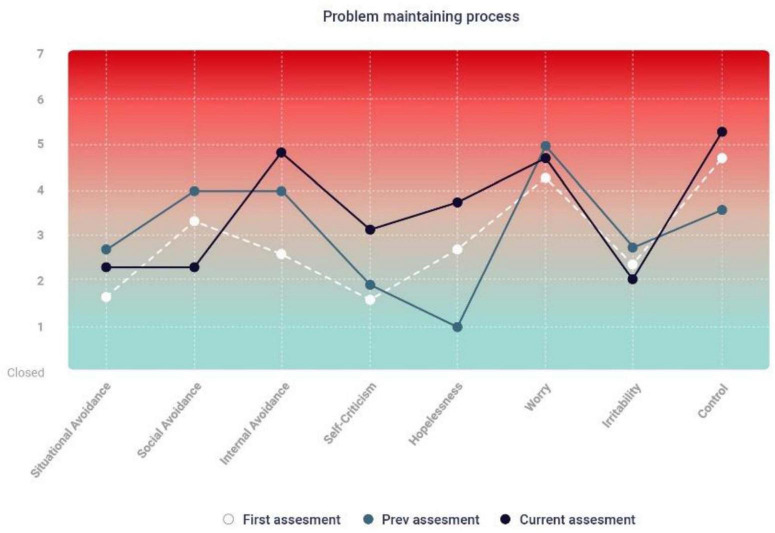
Sonja: feedback report #5 – change and stagnation.

Sonja initially reported a high level of internal distress and pain during therapy. As seen in the clinical feedback report, she continually reported a stable occurrence of struggle for several weeks. The equivalent lines in the graph show the stability of this struggle in her feedback. Her feedback conveyed that her struggle was in the clinical area of distress (high end of the graph) (Figure 2).

The process notes indicate reflections on the need to verbalize this persistent struggle together with Sonja. According to the health record, ‘*the client said that she has difficulty tolerating her feelings. It is hard to name them. It seems important to work with the emotions associated with difficult experiences. Being emotionally close to these experiences and creating meaning around them is considered an important process to the client’* (HRA5). Sonja and the therapist jointly decided that it was important to have an additional focus on internal avoidance. Despite this clinical consideration, we can see in the clinical feedback report several weeks later that the client still had major challenges related to internal avoidance (Supplementary Figure 9).

Although the therapy was focused on this topic, Sonja continued to report a high level of internal avoidance. The process notes included reflection of the therapist on the need to consolidate the meaning of this experience and how internal avoidance could have a direct effect on emotional experiences through therapy. As stated in the health record, ‘…*the client said there were so many things she should have worked on, but that everything seemed so overwhelming. We used psychoeducation for this experience and the development of emotional strength throughout therapy’* (HRA13). Due to the stable feedback reports and the need to address this issue of avoidance, the therapist decided to increase the frequency of future therapy sessions: ‘*the client asked why we meet more often than planned. I explained that I considered her to have more use for weekly sessions (.) to maintain her alliance and therapeutic focus. The client was grateful for this’* (HRA13). The report remained stable for several weeks afterward, before it showed Sonja’s internal pain had worsened (Supplementary Figure 10).

This clinical feedback related to Sonja’s worsening caught the attention of the therapist, who initiated a discussion of it with Sonja. According to the clinical report by the therapist, ‘*We went through the feedback report, (.) and I tried to explore the feedback about how she felt and how she has become significantly worse throughout the therapy. The client explained that this was due to better emotional contact. She said that this was painful, good and important’* (HRA17).

#### Harald

Stagnation in therapy became particularly relevant to Harald. For up to a year and a half, the feedback he reported of his experience of inner pain remained stable (Supplementary Figure 11).

We see from the clinical feedback that Harald experienced minimal symptom relief over a long period. This can be painful for the client and challenging for the therapist. The therapist devoted a considerable amount of time to reflect on the therapy, and if there were issues that were overlooked or something about her practice that should be modified. However, at one point, we can see a sudden significant change in the feedback report (Supplementary Figure 12).

At the start of this session, Harald reported that he did not feel that anything had changed since the last session. Due to the large difference between what Harald conveyed verbally and what he responded to in the clinical feedback system, the therapist chose to comment on the therapy session for that report. According to the clinical report, ‘*We reviewed the report and the client provided input and reflected on the changes. The client expressed excitement because of this and said he thinks it is good to see the progress visually’* (HRB38). The therapist noted in the process notes that Harald’s body language and expression of emotion changed considerably. These positive signs of progress continued for several weeks.

The clinical feedback report showed significant improvement in the following weeks. Harald’s reported struggle changed from clinically severe (black line) to generally mild. One might suspect that the visualization of a concrete improvement may have been the turning point in Harald’s progress and his motivation for further work (Supplementary Figure 13).

### Feedback helped to verbalize the non-verbal

This theme reflected occasions when patients expressed emotionally charged material that seemed to have been partly stimulated by the feedback itself. Many people find it challenging to verbalize difficult experiences and painful feelings. A supportive environment helps many people experience and express these feelings, and this process is one of the central theoretical features of emotion-focused therapy (Greenberg, 2004). The use of clinical feedback was found to be helpful in the process of spotting potential non-verbal changes in the therapy with Sonja and Harald.

#### Sonja

**FIGURE 3:**
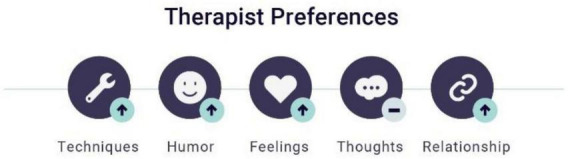
Sonja: feedback report #6 – verbalizing the non-verbal.

During therapy, Sonja used her clinical feedback to report a more holistic impression of her pain. Based on this feedback, the process notes highlighted the therapist’s awareness of this information and her sensitivity to direct changes in the individual session. Before the session, the therapist read the feedback report, which included a clear expression of Sonja’s desire for a greater focus on emotions during therapy (Figure 3).

Having this knowledge of Sonja, the therapist was able to connect this information with the information in the remainder of the feedback report. This awareness influenced the therapist’s sensitivity to potential topics presented with emotional vulnerability. The clinical health report underscored this situation ‘*the conversation centers on her relationship with her mother, an intervention with unfinished business. The client interrupted herself and said it was too much. She said that she felt too much chaos from the emotions inside her. I canceled the intervention and provided psychoeducation about the method instead. The client plans to try again later*’ (HRA6). As described in this report, the therapist incorporated the information from the clinical feedback into her direct clinical expressions during the therapy session, which led to a softening of the ongoing intervention. We can see in Supplementary Figure 14 that Sonja changed her expression of needs in therapy two weeks later.

#### Harald

As stated earlier, Harald’s feedback report on how he was feeling was quite stable; this also applied to his direct contact. It was difficult for the therapist to capture changes based on the clinical impression he gave during the session. However, at one point, the feedback report showed significant worsening in the dimension of situational avoidance (Supplementary Figure 15).

In a conversation with Harald, he talked about a newly experienced incident, in which he was exposed to a sudden encounter that triggered a trauma-related reaction. This was a stressful episode for him, but not clinically apparent to the therapist. With the help of the clinical feedback report, the therapist was able to register the change and verbalize it during the session. The therapist was able to help Harald work through this experience and create meaning in what happened during that particular situation. In this case, the use of clinical feedback made it possible to capture non-verbal and significant changes. Due to this information, the therapist was able to initiate a conversation that validated and reduced the discrepancy between Harald’s cognition and his emotional state of mind (HRB41). After two sessions, Harald reported significantly different feedback related to this incident; it was more similar to the feedback before the incident (Supplementary Figure 16).

### Member checking – clients’ perspectives of the interpretations

The weekly feedback from the clients were interpreted and ‘fed back’ to the clients in different ways. The feedback was communicated through joint conversations about the report, direct dialogue about specific difficulties informed by the report or as indirect information related to the therapeutic process stimulated by the therapist. However, the influence of the therapist and the interpretations were highly relevant in all of these paths of communication. To acquire a better understanding of these processes, Sonja and Harald were invited to participate in a ‘walk-through’ of the study’s results and interpretations with the first author. This step was implemented as an hour-long conversation and interview in the later stages of the analysis, and after both therapies had ended. The aim was to elicit their perspective and feedback on these interpretations and understandings and make them transparent for the reader.

#### Sonja

Sonja told the first author that starting therapy was hard work. She had difficulty establishing trust in other people, including the therapist. As Sonja relayed the different reflections and considerations of the therapist during their meetings, she laughed and suggested that it could not have been easy to establish contact initially with her in therapy. She was convinced that the therapy had been stagnant and seemed meaningless if the therapist did not work explicitly on the alliance. Sonja believed the therapist would have struggled more to help her without the help of the clinical feedback. The feedback was necessary for the client to express her inner struggle and for the therapist to navigate her-unspoken field of pain.

During therapy with Sonja, the therapist focused on Sonja’s internal avoidance. Sonja agrees that this was an important topic, but did not agree with the therapist about the importance of the need to reduce this dimension to achieve symptom-relief. Sonja acknowledged that she still struggled with internal avoidance. Despite this, she had a significantly better quality of life and functioning compared to the period before therapy. She acknowledged that a focus on and an awareness of this challenge had made her more attuned to the personal challenges in everyday life that she must cope with to avoid limiting her options.

Sonja had a good memory of the session in which the interventions were increased and thereby intensified, and she felt the need to withdraw from the therapeutic work. Although she had conveyed that she wanted a greater focus on emotions, she was not prepared for the intervention that followed. She remembered the intervention was intense and unfamiliar, and that it was “too much.” Sonja remembered an explosion of emotions that she was not able to control; thus, she interrupted the work of therapy, and she was still happy that she managed to set this limit. At the same time, this intervention left a deeper mark on her. She reported having inner dialogues with her mother weekly, but still struggled to complete the “conversation” and despairs about this.

Sonja went to therapy for many years during her youth. She has had different experiences with her therapists, and the use of clinical feedback had always been a positive experience, which strengthened the therapy. Throughout her youth, Sonja’s therapists functioned as her caregivers, and this relationship applied to the relationship in question. She acknowledged that the necessary focus on the relationship led her to connect with the therapist. She still remembered the pain she felt when the therapist informed her that their relationship was ending. These feelings were still close to her heart, and tears rolled down her cheeks when she talked about this episode.

#### Harald

Harald told the author that the use of clinical feedback was essential for him to be able to inform the therapist about his inner struggle. He acknowledged his long experience faking “good,” and the use of clinical feedback made it more difficult to continue with this behavior. The experience of giving feedback from a safe place increased his courage to be honest. Harald did not have an explicit memory of extended focus of the therapist on the alliance during the therapy. He revealed that he was highly affected by anxiety and inner tension. He remembered the therapist trying to provide explanations to him, but his stress made it impossible for him to understand the therapist. He also did not dare to speak out because he feared disappointing the therapist and had an inherent desire not to be a nuisance to others. Although Harald’s feedback remained hurtful, he did not consider quitting therapy or changing therapists. He could talk about his fear of changing therapists, which was due to his basic problem with trust and an inner need for secretiveness.

Harald had a good memory of the intervention that was implemented because of having expressed different needs in treatment. When Harald talked about this session, he still experienced physical discomfort. He believed this exercise had a direct impact on the subsequent therapeutic period and the improvement that was documented in the reports. After his greatest shame and pain was “seen” and articulated, he was able to focus on moving forward. Although the therapist considered this therapeutic episode in a different way, both of them considered it important. This outcome underscored the importance of reconnecting verbally after significant breakthroughs and the importance of meaning making to ensure that the client did not feel overwhelmed or disorganized.

Although Harald did not have a clear memory of the different changes discussed in this study, he was glad that clinical feedback was offered to him as part of his therapy. He felt that its use made him more complete. Harald said that having something concrete to look at and visual ‘proof’ that the therapy was working had a positive effect on him.

## Discussion and conclusion

### Discussion

This study examined a course of individual therapy with two clients in which Norse Feedback was used extensively. Information was obtained from journals, feedback reports and process notes to examine these therapies in depth. We have discussed how feedback can influence psychotherapeutic processes by triangulating these sources of information. This study did not aim to present an exhaustive perspective on the use of clinical feedback or to defend or glamourize the use of clinical feedback. In our analyses, certain themes were found to be particularly important. Feedback from the clients actualized the work of alliance formation, concretized changes and stagnation and helped the clients verbalize the non-verbal. One of the three themes was found to be a particularly important factor in the enhancement of the helpfulness of clinical feedback. The use of feedback underscored the importance of collaborative practice in psychotherapy. It was important for the client to receive a response to the feedback they gave; otherwise, giving feedback could have resulted in a destructive experience for the client. Based on the examples from Sonja and Harald, we found that clinical feedback could be a valuable conversation opener, and that it could inform the therapist’s choices. In the following section, we discuss three generalizations of the themes found in the cases presented above: alliance, psychoeducation, and deliberate practice.

### Clinical feedback actualizes the work of alliance formation

How to establish a good alliance has been of clinical interest for decades. Therapists focus daily on how to establish and adjust the alliance so that the client experiences the therapy as safe and meaningful (Muran and Barber, 2011). Bordin (1979) says that the therapeutic alliance consists of three components: the relationship between the client and the therapist, their agreement on tasks and the goal of therapy. He suggests that the alliance is a negotiation process between the therapist and the client, at both the conscious and subconscious levels. Extensive research has shown that the quality of the therapeutic alliance is one of the best predictors of treatment outcomes (Safran and Muran, 2000). However, establishing a good alliance can be difficult, as ruptures may occur. This is a moment of tension or breakdown in the therapeutic alliance (Safran and Muran, 2000). Ruptures can manifest in different ways (Muran and Barber, 2011), and examples of ruptures were observed in the two client-therapist relationships in this case study. In the case of Sonja, the medical health record and reflection notes conveyed an impression of an extended focus on alliance formation. Despite this, Sonja gave direct feedback that she did not feel safe with the therapist. In this case, the alliance was not sufficiently established, and further reaction and therapeutic work following this statement may have been crucial for repairing the alliance. In the case of Harald, the rupture was presented visually through written weekly feedback stating that he did not feel safe or understand the tasks or goals of therapy. This alliance was weak for several months. It is reasonable to assume that he began to lose trust in the efficacy of therapy. The likelihood that this prolonged rupture would result in a dropout was high. Clients who experience ruptures may react by either withdrawing from therapy or confronting the therapist. Ruptures often occur when the therapist provides interventions for which the patient has not been sufficiently prepared to receive them, as described by Coutinho et al. (2011). This description exemplifies the episodes of Sonja and Harald. In both situations, the client gave feedback that conveyed a wish for an increased focus on emotions. In the case of Sonja, she interrupted the intervention and replied verbally that she did not want to explore this topic further. In the case of Harald, he stayed in the intervention, but acknowledged afterward that it was too overwhelming, which resulted in his withdrawal of emotion in subsequent sessions. These alliance ruptures may have been caused by the therapist attempting to move too quickly toward difficult topics, need for additional coping skills, or several other issues.

Establishing a therapeutic alliance can be challenging, and every client has individual needs and preferences. A recent meta-analysis by Lavik et al. (2018) on early alliance-formation processes from the perspective of therapists and clients, found that therapists and clients had different needs and considerations. The clients emphasized the need for a warm and competent therapist, who had a holistic approach to individuals and their difficulties. They needed to feel safe and supported regarding their therapy and hopes for the future. The clinicians had a broader perspective on alliance formation. With the focus on therapeutic safety, a desire to establish hope and understanding and strengthen the clients’ agency, they worked on balancing the therapeutic tone with clinical interventions and body language (Lavik et al., 2018). This approach exemplified the therapy with Sonja, where the therapist tried to use a challenging evidence-based practice of emotion-focused treatment while balancing warmth and support. Despite this, Sonja’s clinical feedback showed that these attempts did not work. A similar situation was observed in the therapy with Harald, although to a greater extent. This observation emphasizes that alliance formation is a personal process, in which the therapist must sense and adjust to the needs and preferences of the individual client. In both of these examples, the use of clinical feedback made this struggle more concrete and manageable. It made it easier to include lack of connection as an explicit topic in therapy, which was exemplified in both therapies and seems to have influenced the processes of repairing the rupture and re-connection.

The knowledge from the above meta-analysis and the two clinical cases shows how difficult it can be to establish a good alliance, and how easy it can be to experience an alliance rupture. There were several situations in both therapies, where the therapist came very close to a rupture, and was unaware of it. Although we strive for an equal relationship, we can easily imagine that telling your therapist that you do not feel safe or do not agree with the therapeutic work can be difficult. Fear of hurting another person or of rejection might influence one’s behavior. Nevertheless, in both cases, our two clients overcame these barriers, and the clinical feedback was an important factor in this process. A clinical instrument can help navigate sensitive and complex areas and support the clinician’s attempt to balance the therapy and repair a ruptured alliance, if necessary. Research has shown that dyads who repair ruptured alliances often end up with a stronger alliance than the alliance before the rupture (Safran and Muran, 2000; Katzow and Safran, 2007) and have improved outcomes (Eubanks et al., 2018).

### Clinical feedback as psychoeducation

There is no universal definition of psychoeducation (Gordon et al., 2015), but it can be defined as ‘…*an intervention with systematic, structured and didactic knowledge transfer for an illness and its treatment, integrating emotional and motivational aspects to enable patients to cope with the illness and to improve its treatment adherence and efficacy*’ (Ekhtiari et al., 2017). Psychoeducational interventions are used in the health service for a wide range of patient groups. Self-help courses (including psychoeducation) for various difficulties are free of charge on the internet. Psychoeducation can also be used in the context of a clinical feedback system. Initially in therapy, it is important that the therapist understand the client’s struggle. The use of clinical feedback can provide the therapist with a large amount of information – not only in relation to the client’s difficulties, but also to the surrounding circumstances that may affect the expression of pain and how best to work clinically with it. Sonja and Harald gave the impression that they found the use of clinical feedback as a helpful tool to express the extent of their pain. Combining the elements of psychoeducation and information from clinical feedback may be more potent compared to general psychoeducation. By using this resource, the therapist can actualize the information in a more individual and sensitive way. By connecting these two approaches, clients’ should acquire a better understanding of how their difficulties affect aspects of their living situation. Research conducted with persons with severe diagnoses, such as schizophrenia and bipolar disorder found that the use of psychoeducation had a positive effect on the individual’s prognosis and psychosocial functioning (Orm et al., 2020). As seen in the therapy with Sonja and Harald, the use of feedback increased their understanding of how their struggles affected their feelings and daily lives, and how to regain agency during the recovery process.

Psychoeducation is a well-known technique in specialist care settings in Norway and in general health care and community settings. Studies have shown that psychoeducation is effective in prevention and quality improvement programs (Dowrick et al., 2000). The use of clinical feedback in settings with different levels of health care, can provide communication support for both clients and their helpers, who, in turn, can provide a more holistic and cooperative approach. However, Orm et al. (2020) argue that psychoeducation is not exclusively positive regardless of the clients’ problems. Psychoeducation alone will not treat the basic problem, but it may empower the client sufficiently to understand how to handle and work with their difficulties. By personalizing psychoeducation, agreement on the tasks and goals of therapy could potentially be more effective and preparatory. Personalized psychoeducation can equip clients for therapeutic interventions and reduce the likelihood of an alliance rupture. The health records of the two individuals presented in this study provide examples of how to connect information from clinical feedback with psychoeducation. When using feedback as a psychoeducational tool, it is important to be aware of the specific purpose of the psychoeducation and provide psychoeducation in a non-stigmatizing way (Øverland and Zahl, 2021).

### Supervision and deliberate practice

Clinical feedback can be applied in different ways. As shown in this case study, the role of clinical feedback systems was natural in the therapy process. Such a system can also be used for supervision. In the same way that the patient’s feedback can influence therapy, it can contribute supplementary information to discussions with the supervisor. The therapist, who conducted the two therapies, had weekly supervision. Reflective notes describe discussions about the clinical processes of feedback reports and video-recordings (the latter only with Sonja). Furthermore, one can use the feedback to explore potential blind spots of the therapist. In situations of stagnation, one may wonder whether the therapist has conscious or unconscious vulnerabilities that make it difficult to move forward with the patient’s therapy. In the case of Harald, one might wonder if it was best to continue with the therapy, instead of offering another therapist. Could maintaining the process be destructive to the client? In such situations, it is conceivable that clinical feedback can be used to facilitate deliberate practice.

Deliberate practice is defined as “*individualized training activities, specially designed by a coach or a teacher to improve specific aspects of an individual’s performance through repetition and successive refinement”* (Ericsson and Lehmann, 1996). The main goal of deliberate practice is to improve performance. It is a highly structured activity consisting of repeated experiences of critical aspects of an issue under discussion, with feedback from a supervisor (Ericsson et al., 1993). Deliberate practice has been used in psychotherapy supervision and training programs by using ROM-CF tools to identify how therapist performance can be evaluated and improved (Goldberg et al., 2016). Research has shown that the amount of time therapists use to improve their therapeutic skills (e.g., mentally running through and reflecting on past and future sessions), has a significant effect on the client’s outcomes (Chow et al., 2015). As suggested by Hill et al. (2017), expert therapists prioritize large amounts of time in their deliberate practise. This includes supervision, pondering clients between sessions and preparing for concrete interventions. One might wonder whether the therapy with Harald might have progressed faster if the therapist had also used deliberate practice during supervision, in addition to personal descriptions and clinical feedback. Harald was offered a new therapist, but he declined the offer. Luckily, the courage and perseverance of both Harald and his therapist resulted in a good outcome, despite undergoing a tough process.

### Limitations

This research is a clinical case study of two clients in therapy. Because of the limited selection of participants, the results cannot be generalized to a broader range of populations. However, this case study still has value in developing hypotheses, in particular, by engaging clinicians in professional discussions about the clinical aspects of ROM and clinical feedback. One apparent limitation is that we did not have systematic data on which therapeutic interventions were given in individual sessions. As such, analyses regarding the themes in the context of specific interventions, such as chair-work, rather than the session as a whole, cannot be undertaken. The reason for this was that the study was conceived of as client-centered rather than intervention-centered from the inception. The same therapist conducted individual therapy with both clients, and therefore the analysis may over-represent the therapist’s perspective relative to the patients’. We worked reflexively with this issue, and believe that this potential risk is mitigated by the other co-authors being more independent and experienced researchers and data analysts, and by the implementation of the client member checking procedure. Additionally, all authors also contributed to the development of the clinical feedback system used with these clients. Due to their involvement, it is necessary to emphasize that both of these factors are likely to have an influence on the therapy and the presentation of findings. This study contributes to the generation of hypotheses of how feedback can support therapy when it is wanted and used. The authors would like to state explicitly that although the concepts presented in this paper are positive with regard to this clinical tool, our intent is not to claim that, based on these results, clinical feedback is always good, or that Norse Feedback is a preferable system. The study is not a critical examination of such questions, but rather an exploration of a selection of potentially constructive processes. The choice of clients has also influenced the findings. Both of the clients who participated in this study were fond of using the clinical feedback system. If we had invited different clients, different themes and discussions might have emerged.

## Conclusion

Clinical feedback can be a positive supplement to the therapeutic work and process. The importance of making this as a joint tool between the client and the therapist is significant. Clinical feedback alone does not guarantee a good therapeutic process, and it is important to emphasize the necessity of daring to reflect on blind spots and shortcomings that need supervision.

## Data availability statement

The datasets presented in this article are not readily available because this is clinical case materials. Requests to access the datasets should be directed to MH, marianne.magnesdotter.helleseth@helse-forde.no.

## Ethics statement

Ethical approval was not required for the studies involving humans because this project does not involve a data collection for research purposes. Both participants have given their fully informed written consent to the case study, and they have been presented the manuscript for comment and confirmation of this consent prior to submission for publication. Participants’ personal information has been anonymized. The studies were conducted in accordance with the local legislation and institutional requirements. The participants provided their written informed consent to participate in this study. Written informed consent was obtained from the individual(s) for the publication of any potentially identifiable images or data included in this article.

## Author contributions

MH collected and organized the data and led the work on analyses and writing. AM contributed to writing the manuscript. CM contributed to data analyses and writing the manuscript. All authors contributed to the article and approved the submitted version.
