# Supplementary material for: Case report: A dual case study of how clinical feedback can be a communication aide and influence therapeutic work
Source: Front Psychol. 2023 Dec 13;14:1199431. doi: 10.3389/fpsyg.2023.1199431 (PMC10752420; doi:10.3389/fpsyg.2023.1199431)

**Online supplement file**

**Supplementary Fig. 1** Sonja: Feedback report #3 – alliance


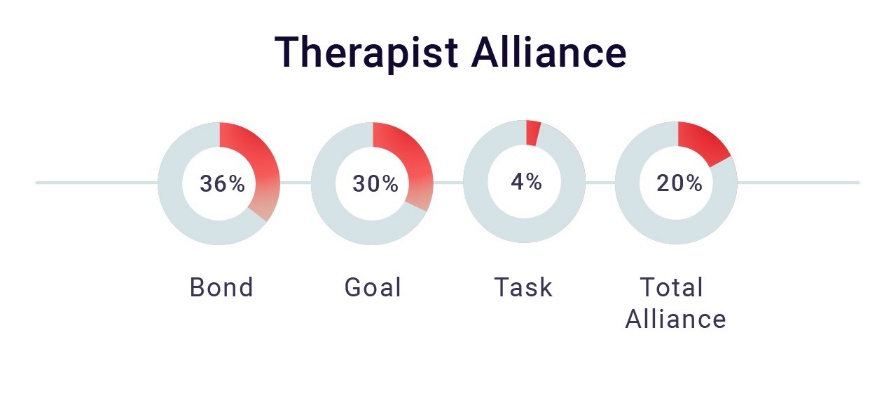


**Supplementary Fig. 2** Sonja: Feedback report #9 – alliance


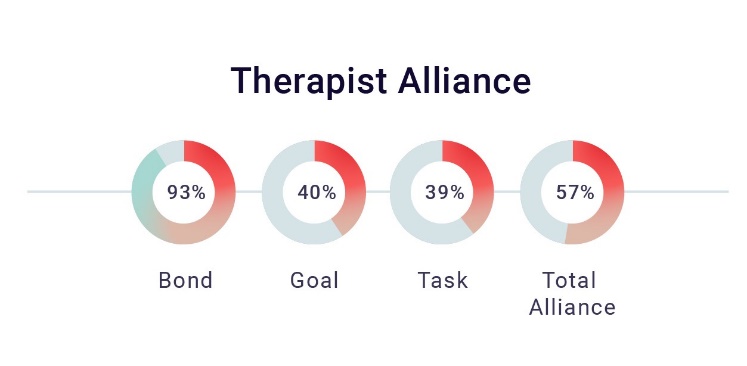


**Supplementary Fig. 3** Sonja: Feedback report #10 – alliance


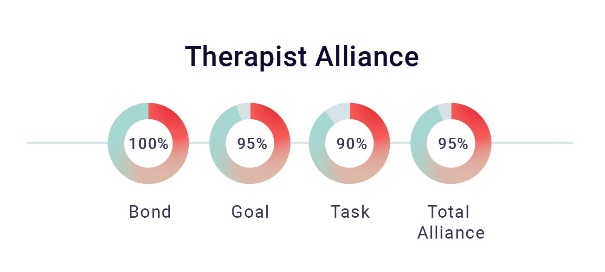


**Supplementary Fig. 4** Harald: Feedback report #9 – alliance


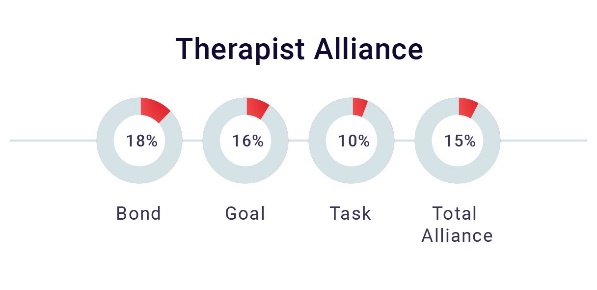


**Supplementary Fig. 5** Harald: Feedback report #11 – alliance


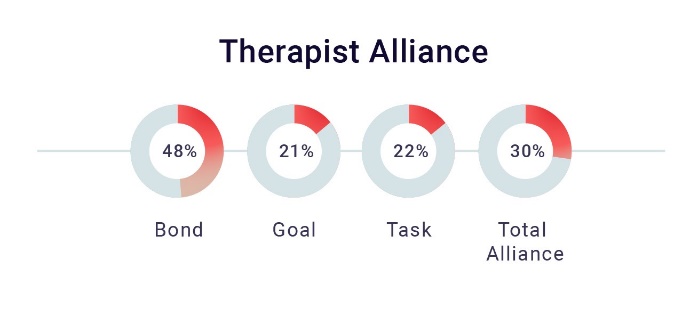


**Supplementary Fig. 6** Harald: Feedback report #17 – alliance


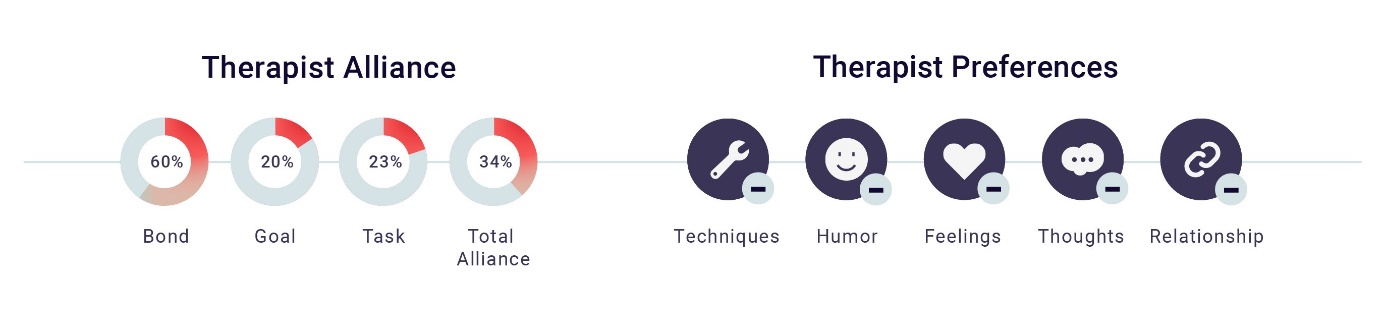


**Supplementary Fig. 7** Harald: Feedback report #18 – alliance


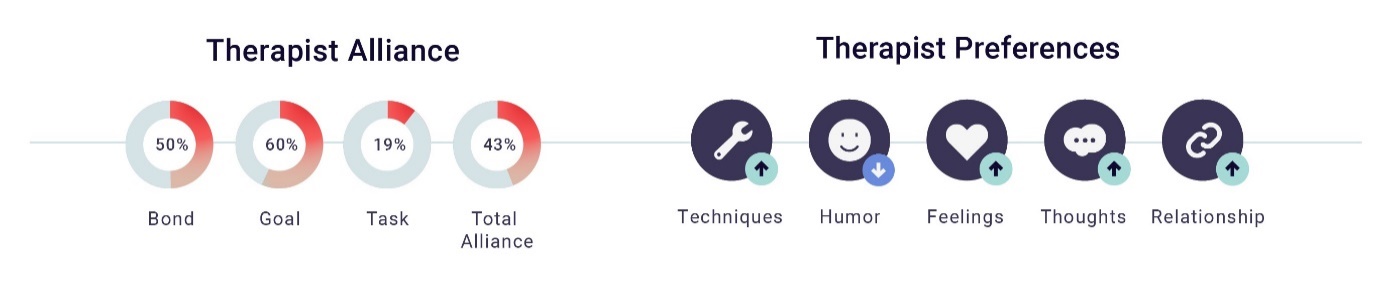


**Supplementary Fig. 8** Harald: Feedback report #21 – alliance


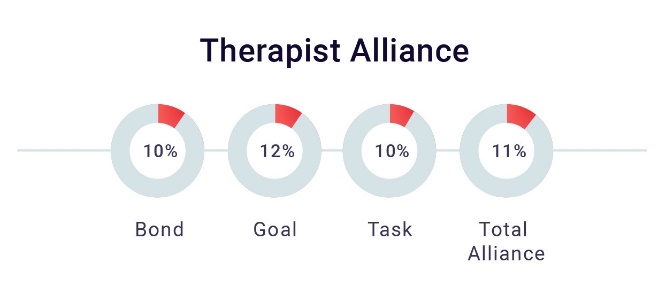


**Supplementary Fig. 9** Sonja: Feedback report #11 – change and stagnation


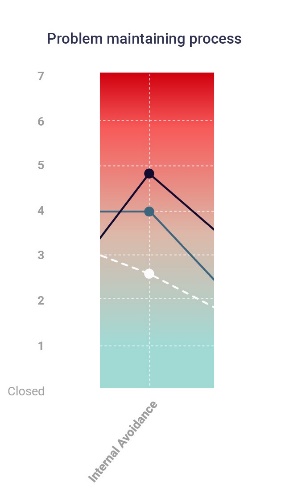


**Supplementary Fig. 10** Sonja: Feedback report #13 – change and stagnation


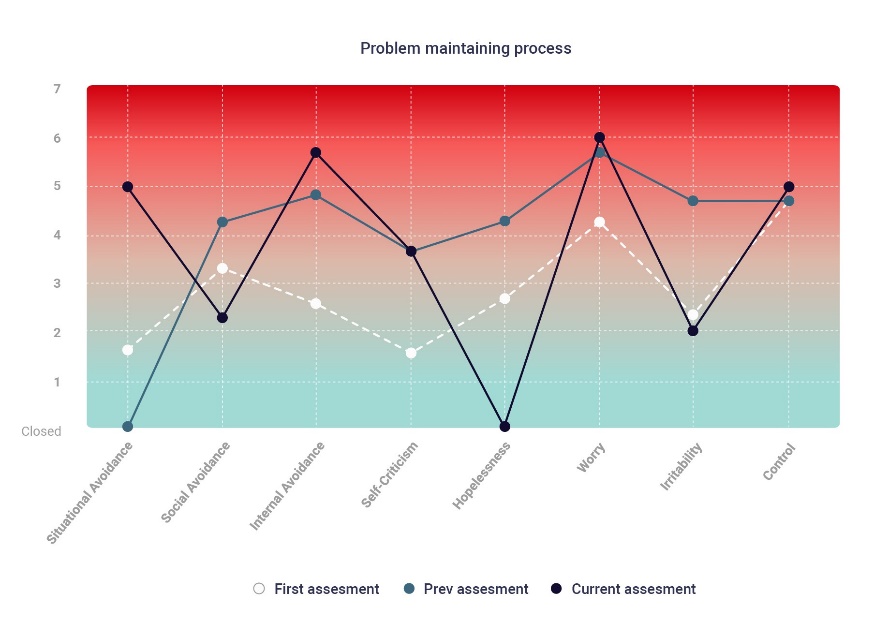


**Supplementary Fig. 11** Harald: Feedback report #26 – change and stagnation


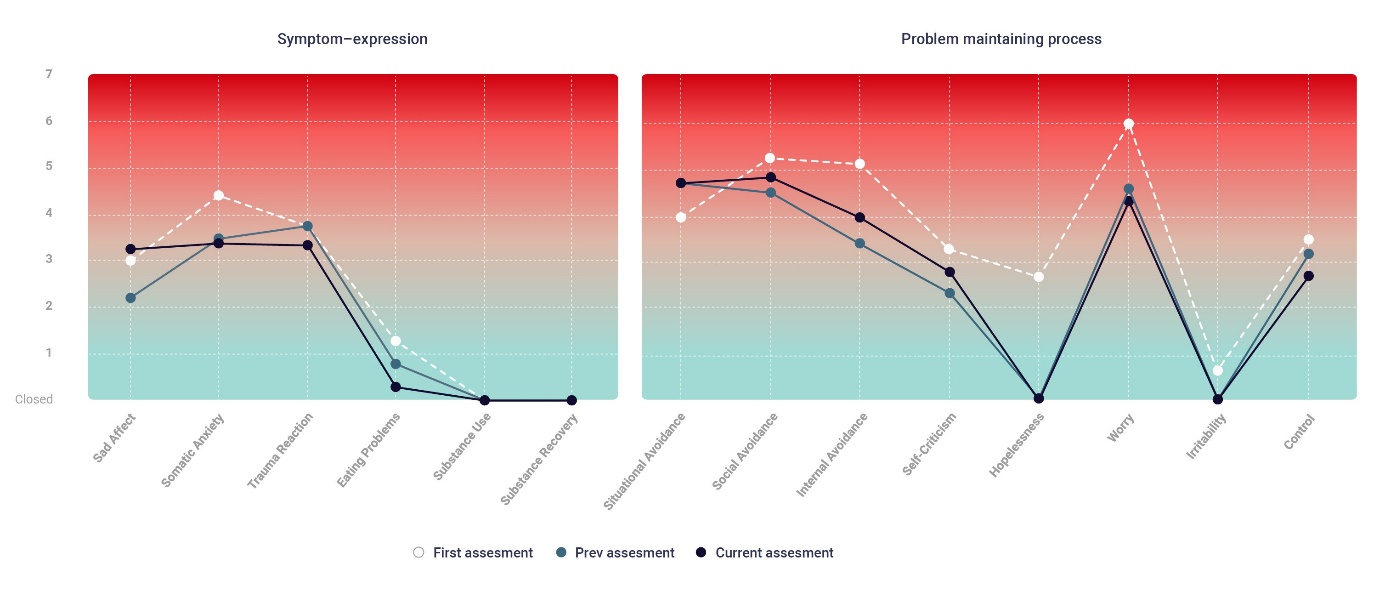


**Supplementary Fig. 12** Harald: Feedback report #27 – change and stagnation


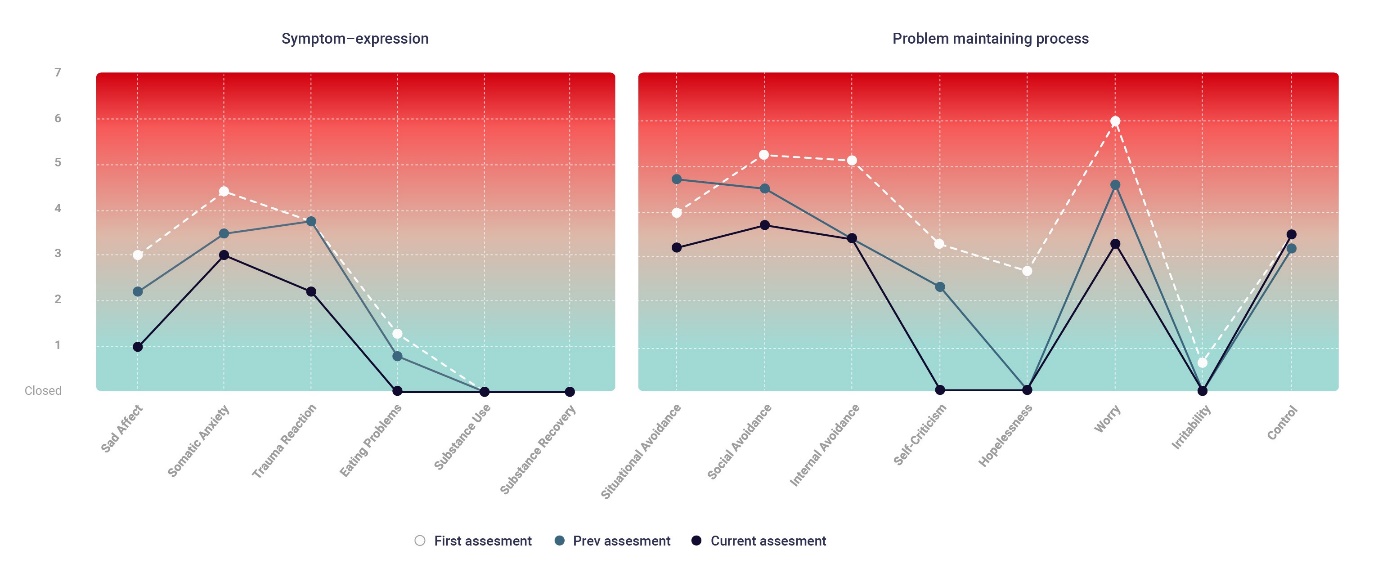


**Supplementary Fig. 13** Harald: Feedback report #33 – change and stagnation


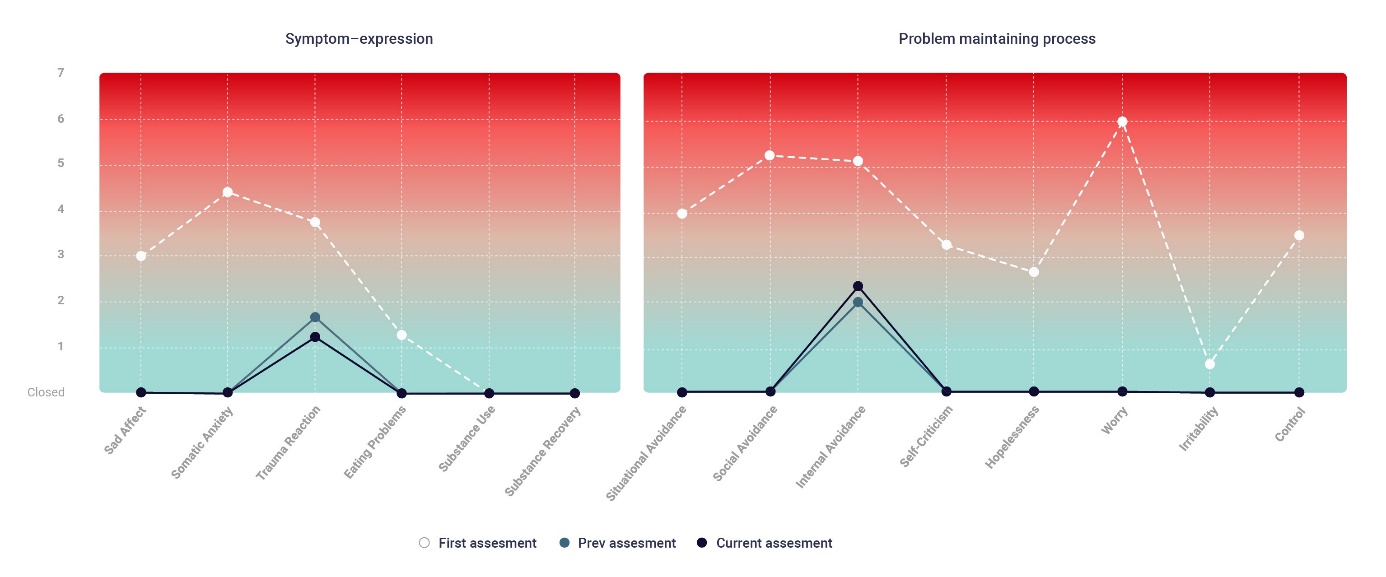


**Supplementary Fig. 14** Sonja: Feedback report #8 – verbalising the nonverbal


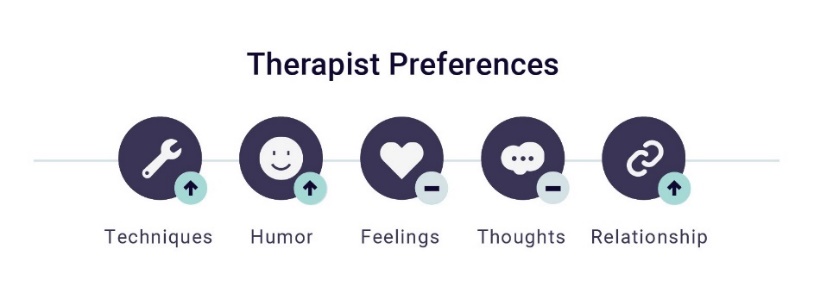


**Supplementary Fig. 15** Harald: Feedback report #30 – verbalising the nonverbal


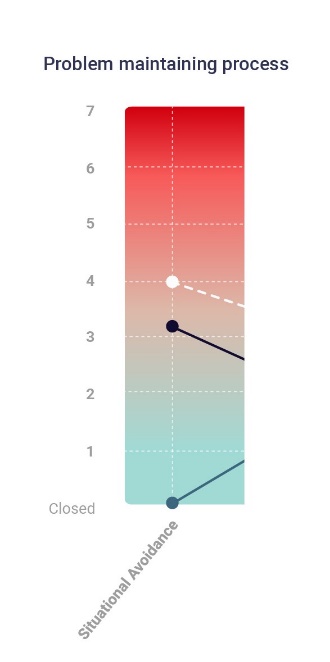


**Supplementary Fig. 16** Harald: Feedback report #32 – verbalising the nonverbal


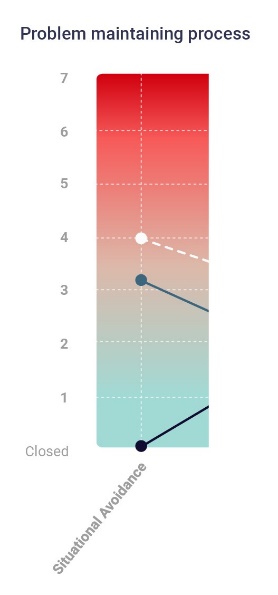

Supplement: Supplementary file 1 [file Data_Sheet_1.docx]
